# Blood Vessel Matrix Seeded with Cells: A Better Alternative for Abdominal Wall Reconstruction—A Long-Term Study

**DOI:** 10.1155/2015/890613

**Published:** 2015-02-05

**Authors:** Maciej Nowacki, Arkadiusz Jundziłł, Łukasz Nazarewski, Andrzej Kotela, Tomasz Kloskowski, Joanna Skopińska-Wisniewska, Magdalena Bodnar, Aleksander Łukasiewicz, Sławomir Nazarewski, Ireneusz Kotela, Marek Kucharzewski, Marta Pokrywczyńska, Andrzej Marszałek, Tomasz Drewa

**Affiliations:** ^1^Chair of Regenerative Medicine, Department of Tissue Engineering, Collegium Medicum, Nicolaus Copernicus University in Torun, Ulica Karlowicza 24, 85-092 Bydgoszcz, Poland; ^2^Department of General, Transplant and Liver Surgery, Warsaw Medical University, Ulica Banacha 1A, 02-097 Warsaw, Poland; ^3^Department of Orthopaedics and Traumatology of Musculoskeletal System, 1st Faculty of Medicine, Medical University of Warsaw, Ulica Lindleya 4, 02-005 Warsaw, Poland; ^4^Department of Chemistry and Photochemistry of Polymers, Faculty of Chemistry, Nicolaus Copernicus University in Torun, Ulica Gagarina 7, 87-100 Torun, Poland; ^5^Department of Clinical Pathomorphology, Collegium Medicum, Nicolaus Copernicus University in Torun, Ulica Skłodowskiej Curie 9, 85-094 Bydgoszcz, Poland; ^6^Department of General, Vascular, and Transplant Surgery, Warsaw Medical University, Ulica Banacha 1A, 02-097 Warsaw, Poland; ^7^Department of Orthopedic Surgery and Traumatology, Central Research Hospital of Ministry of Interior, Ulica Wołoska 137, 02-507 Warsaw, Poland; ^8^Institute of Physiotherapy, The Jan Kochanowski University of Humanities and Sciences, Al. IX Wieków Kielc 19, 25-317 Kielce, Poland; ^9^School of Medicine with the Division of Dentistry in Zabrze, Chair and Department of Descriptive and Topographic Anatomy, Medical University of Silesia, Ulica Jordana 19, 41-808 Zabrze, Poland; ^10^Department of Urology, Nicolaus Copernicus Hospital in Torun, Ulica Batorego 17/19, 87-100 Torun, Poland

## Abstract

*Purpose*. The aim of this study was to present abdominal wall reconstruction using a porcine vascular graft seeded with MSC (mesenchymal stem cells) on rat model.* Material and Methods*. Abdominal wall defect was prepared in 21 Wistar rats. Acellular porcine-vascular grafts taken from aorta and prepared with Triton X were used. 14 aortic grafts were implanted in place, of which 7 grafts were seeded with rat MSC cells (Group I), and 7 were acellular grafts (Group II). As a control, 7 standard polypropylene meshes were used for defect augmentation (Group III). The assessment method was performed by HE and CD31 staining after 6 months. The mechanical properties have been investigated by Zwick&Roell Z0.5.* Results*. The strongest angiogenesis and lowest inflammatory response were observed in Group I. Average capillaries density was 2.75, 0.75, and 1.53 and inflammatory effect was 0.29, 1.39, and 2.72 for Groups I, II, and III, respectively. The means of mechanical properties were 12.74 ± 1.48, 7.27 ± 1.56, and 14.4 ± 3.7 N/cm in Groups I and II and control, respectively.* Conclusions*. Cell-seeded grafts have better mechanical properties than acellular grafts but worse than polypropylene mesh. Cells improved mechanical and physiological properties of decellularized natural scaffolds.

## 1. Introduction

Ventral hernia continues to be a fundamental problem for surgeons worldwide. It occurs in 1% of patients with primary wound healing, 11% of patients with postoperative wound infection, and up to 20% of all patients undergoing abdominal surgery in a long-term follow-up [1–5]. This abdominal wall defect is also an emerging problem in new born infants, which mainly is correlated with omphalocele or gastroschisis [6, 7].

Throughout the years, many techniques have been described for the repair of a ventral hernia; however, none of them has gained universal acceptance or preference. Nowadays, tension-free closure of the abdominal wall with the use of prosthetic mesh material is the most commonly performed procedure [8, 9]. Despite many advantages, several problems connected with implantation of prosthetic mesh are reported. Currently available synthetic materials are suboptimal and may lead to many unacceptable complications. Most of them are related to chronic inflammatory response caused by the mesh and resulted in adhesions, enterocutaneous fistulae and scar formation, high infection rates, and chronic pain [4, 8–13]. In previous studies we used polyglycolic acid (PGA) scaffold seeded with 3T3 fibroblast or collagen slices for abdominal wall reconstruction [14, 15]. Promising results encourages us to continue this experiment. In 2008, Bellows et al. showed effectiveness of decellularized matrix generated from blood vessels of swine in abdominal wall reconstruction [16, 17]. In our study we go one step further by seeding scaffold with mesenchymal stem cells (MSC) derived from bone marrow.

The important aspect in the development of this specific clinical problem seems to be the use of regenerative medicine methods based on the achievements in tissue and biomaterial engineering.

In our study, commonly used polypropylene mesh was compared with decellularized porcine aortic MSC unseeded and seeded grafts, in the reconstruction of rat abdominal wall defect in terms of effectiveness, strength, adhesion formation, histological changes, and complication development.

## 2. Materials and Methods

### 2.1. Animal Model and Surgical Procedure

21 male, 10-week-old Wistar rats from one strain were selected for this study. All animals had comparable weight oscillating between 180 and 200 grams. Animals were divided into three equal groups (7 rats for each group). After anesthesia, the skin was cut in the middle line, and then both sides were carefully prepared to expose the fascia and the muscle layer. The anterior abdominal wall defect was prepared by cutting a rectangle flap (3 cm × 2 cm). All used graft types and control mesh were sawn in a place of artificial defect using 4-0 nonabsorbable, monofilament, and polyamide sutures which served as a marker (Figure 2). Animals were separated in individual cages. Rats were not given any medication except 2-day supply period of nonsteroidal anti-inflammatory drugs (ibuprofen) after surgery. Follow-up period was 6 months. The Local Ethical Committee permission was obtained for the experiment (number 28/2009).

### 2.2. Grafts Used in Experiment

We have used porcine-vascular grafts taken from thoracic aorta (descendent part) and prepared as an acellular matrix with Triton X-100 using the method described by Gamba et al. in 2002 [18]. 14 aortas acellular tissue matrix (ACTM) grafts were carefully and precisely sawn in place of defect, 7 of which were previously (7 days before) seeded with rat MSC (Group I) and 7 were acellular grafts (Group II). The MSC were taken from the primary cell culture established from the femoral bone marrow of an 8-week-old Wistar rat and cultivated in standard environment incubation (37°, 5% CO_2_). As a control (Group III) 7 standard polypropylene meshes were used for augmentation. Graft was seeded with density of 1 × 10^6^ cells/cm^2^. Cells were seeded using modified method described by Drewa et al. [13]. Briefly, graft was placed on plate with small amount of medium and an initial number of 3 × 10^5^ cells/cm^2^ were seeded using small drops to avoid cell migration outside the graft. After medium with cells being soaked through the graft (about 6 hours), this step was repeated until an initial number of 1 × 10^6^ cells/cm^2^ were reached. Grafts were seeded with cells one week before transplantation.

### 2.3. Adhesion Evaluation

Quality of peritoneal adhesions was measured using Jenkins scale (0: lack of adhesion; 1: minimal adhesions easy to separate; 2: moderate adhesions hard to separate; 3: dense adhesions that could be separated with sharp tool). Results in Table 1 are presented as mean of values obtained from particular groups. The incidences of peritoneal adhesions in all tested groups are presented in Figure 3.

### 2.4. Mechanical Properties

The mechanical properties have been investigated with Zwick&Roell Z0.5 machine. A tensile strength (tensitometry) was performed using specimen of biomaterial-native tissue union. Samples were cut into 3 similar segments and evaluated separately. The samples were fixed in tensiometer clamps and the stretching was performed with speed 250 mm/min to the breaking point.

### 2.5. Histology and Immunohistochemistry

All samples were fixed in 10% buffered formalin for 24 h and processed for routine paraffin embedding. Five *μ*m thick sections were obtained from paraffin-embedded samples and stained with HE for further evaluation. Histological sections were analyzed semiquantitatively according to the following scoring system: for inflammatory infiltration composed of T-lymphocytes, plasma cells, and macrophages (0, 1+, 2+, and 3+: absence, minimal infiltrates, infiltrates present in aggregates, or follicles formation, resp.).

Capillary density was measured and presented as average number of vessels <20 *μ*m in diameter per field 500–400 *μ*m. Capillaries density scores 0, 1, 2, and 3 corresponded, respectively, to absent, low (<5 vessels), moderate (5–8 vessels), and high (>8 vessels).

Adhesion-carrying tissues were excised* en bloc* with biomaterials and fixed in 10% buffered formalin. Sections with a thickness of 5 *μ*m were stained with hematoxylin-eosin for light microscopy to evaluate the structure of the connective tissue and the healing process.

Angiogenesis was analyzed using immunohistochemistry method. Tissue specimens or single cells solutions were fixed with 7% formaldehyde before analysis. Tissue slides pieces (4 *μ*m thick) were deparaffinized, rehydrated, and washed in distilled water. Antigenic determinants were exposed by heating in citrate buffer (pH = 6) in microwave or EDTA buffer (pH = 8) in water bath. Incubation in 3% H_2_O_2_ (RT) inhibited endogenous peroxides activity. Nonspecific binding of antibodies was blocked by addition of 5% BSA (Sigma, Germany). Tissue slides were then incubated with primary monoclonal antibodies. In the next stage incubation with secondary antibodies was performed (DAKO EnVision TM + System Labelled Polymer HRP + Anti Mouse, DAKO, Denmark). Antigen-antibody complexes were visualized using 3,3′-diaminobenzidine (DAB(+) Chromogen, DAB(+) Substrate Buffer, DAKO, Denmark). Nucleus visualization was performed using hematoxylin staining, dehydration, radiography, and closing in Canadian balsam. Level of analyzed markers expression was established on the basis of 12-point IRS scale (immunoreactive score) by Remmele [6]. Angiogenesis was assessed with CD31 expression.

### 2.6. Statistical Analysis

Statistical analysis between all three tested groups was evaluated using Student's *t*-test or Cochran-Cox test. The significance level *P* < 0.05 was used as reliable.

## 3. Results

### 3.1. Before Implantation

**Figure 1:**
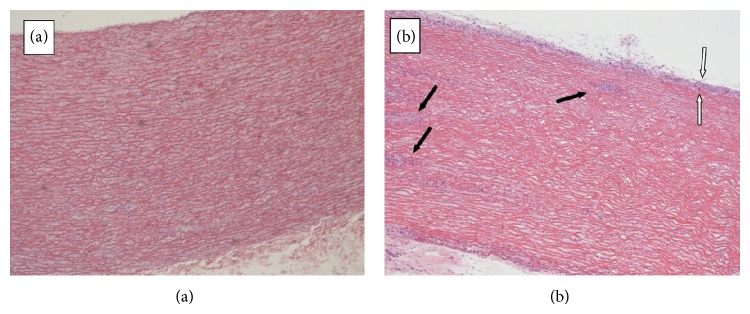
Porcine-vascular grafts. (a) Acellular graft after decellularization in Triton X-100, lack of cell layers can be observed. (b) Acellular graft seeded with MSC after 1 week* in vitro* culture, cell layers covering graft surface (between white arrows) and cell clusters inside the graft (black arrows) can be observed. Light microscopy, magnification 4x.

Acellular structure of vascular graft after Triton X-100 treatment was confirmed using HE staining. Cell layers on graft surface and cell clusters within graft were observed after 1 week of* in vitro* culture of mesenchymal stem cell on the vascular graft (Figure 1).

### 3.2. Surgical Procedure

All animals survived the scheduled assessment period. There were no complications like hernia, fistula, or need for antibiotic therapy associated with the infection.

### 3.3. Adhesion Evaluation

**Figure 2:**
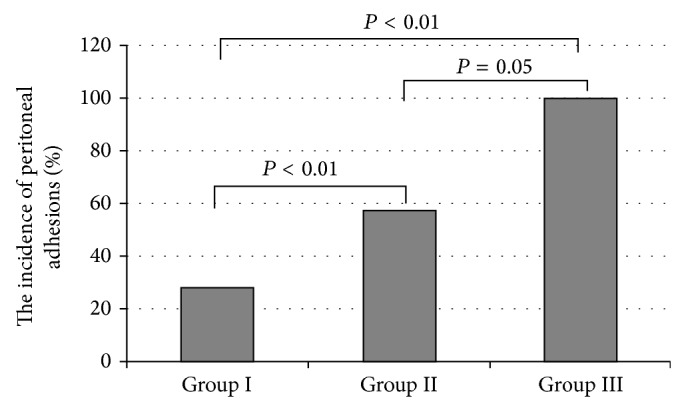
The incidence of peritoneal adhesions in three tested groups.

The lowest number of peritoneal adhesions was observed in Group I (Figure 2). These adhesions had the lowest quality in Jenkins scale (Table 1). In Group III, adhesions were observed in all cases, and their quality was the best in Jenkins scale from all tested groups.

### 3.4. Mechanical Properties

Biomaterial seeded with MSC promotes a robust and durable alloplast-soft tissue combined with great adaptability to the abdominal wall (Group I). Despite great adaptability and low inflammation in adjacent tissue (Group I), the best durability and tensile strength with low modulus of elasticity was observed in Group III (control group). The average tensile strength was higher for scaffold seeded with MSC compared to unseeded scaffold and comparable to control group (Table 1). We used only one time point to measure grafts properties in order to obtain sufficient strong adhesion with surrounding tissues which enabled reliable results of tensile strength test. We consider 6 months to be sufficient observation time on rat model. Additionally authors wanted to ensure that result of this experiment would provide significant data about success for this type of therapy.

### 3.5. Histology and Immunohistochemistry

**Figure 3:**
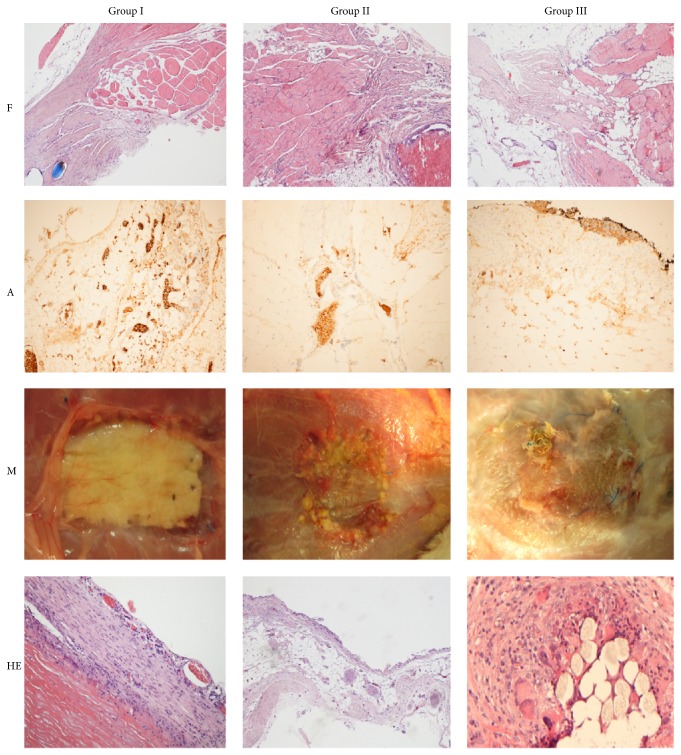
Fusion between native muscle tissue and graft (F); angiogenesis within the graft (A); macroscopic evaluation (M) and inflammation (HE) in all three experimental groups after 180-day follow-up. Light microscopy, magnification 10x.

Light microscopy evaluation indicated the strongest inflammatory healing by fibroid reaction and scar formation in control group (Figure 3). After abdominal wall defect augmentation with cell-seeded graft spontaneous vascularization in relation to low inflammatory wound healing response induced by surgical proceeding without biomaterial resorption was observed (Figure 2). Contrariwise acellular vascular graft resorption in Group II was observed in alloplast-muscle tissue union place as destruction.* Ex vivo* mechanical characterization (tensiometry) acknowledged this observation. Abdominal wall defect reconstructed with polypropylene mesh did not shrink, came loose, or migrate, as well as acellular matrix augmented with cultured cells.

Histological sections in Groups I and II demonstrated extensive angiogenesis in whole implanted biomaterial, prevailing in Group I. The average capillaries density prevailed in Group I resembled fully developed vessels estimated by CD31 expression. The capillary density and number of new vessels were the highest in Group I (Table 1). The histopathological assessment clearly indicated that MSC seeded on acellular vascular scaffold have acceptable impact on formation of new blood vessels which further organize into branched microvascular network fully integrated with host (Figure 3).

The strongest inflammatory effect was observed in polypropylene mesh group. In group augmented with cell-seeded scaffold only mild inflammatory effect was noticed. The group with unseeded scaffold demonstrated almost full resorption due to inflammatory process (Table 1).

### 3.6. Statistical Analysis

All differences in obtained results were significantly important (*P* < 0.05) except results from mean tensile strength between Group I and Group III (*P* = 0.1).

## 4. Discussion

Several studies have been conducted in order to find a new reconstruction material that would minimize the risk of complications. In consequence, many investigators focus on the use of biologically derived materials (e.g., fascia flaps, collagen membranes) and culture-seeded synthetic materials [13, 14, 19]. Those materials reveal capacity to induce milder inflammatory response, improve angiogenesis, enhance cell migration, and protect from infection [18, 20–23]. It was demonstrated that biological properties of implanted cell-seeded grafts result in better outcome [13]. In this study, we choose MSC derived from bone marrow. Adipose derived mesenchymal stem cells (ADSC) are also promising source of stem cells because of easy isolation and culture procedures properties. Properties of this cell type are still inadequately studied. Several papers call into question that ADSC can be differentiated into muscle. In comparison to bone marrow MSC and ADSC are heterogeneous cells, because fat tissue can be collected from different part of body. Researchers obtained different results using ADSC, depending from the location from which fat tissue was collected [24–26]. Therefore, MSC derived from bone marrow which is more homogenous cell line with confirmed properties to differentiate into muscle cells were better option in this study [27].

Extracellular matrices are obtained from skin, facial structures, small intestine submucosa, and porcine blood vessels. The last source seems to be unique due to natural 3D structure of collagen and elastin of blood vessel wall. This kind of engineered tissue has been already investigated in many studies in terms of adhesion formation, angiogenesis, and biomechanical properties and served as a good option for the repair of abdominal wall tissue defects [13, 14]. However, there is lack of experiments that use a combination of aortic decellularized graft seeded with mesenchymal stem cells.

Naturally derived vascular grafts consist of proteins, glycosaminoglycans, various collagen types (I, III, IV, VI, VIII, XV, and XVIII), laminins, elastin, fibrillin, proteoglycans, vWF, and other components which support cell adhesion, migration, and proliferation [28, 29]. This feature makes naturally derived vascular grafts more suitable for tissue engineering approach than textile implants. Vascular grafts pose highly porosity structure, especially after decellularization process (99.93% porosity with average pore diameter 14.2 *μ*m compared to 72.51% and 11.3 *μ*m of native tissue) [30]. This porosity should be enough for cell growth inside the matrix.

Very often consequences of increased adhesion formation include subsequent life-threatening intestinal obstruction and perforation. Polypropylene mesh was noted to cause adhesions that covered over 70% of the implanted material [14], whereas animal studies evaluating adhesion to various biologic matrices have found a significant reduction in the number and severity of these harmful adhesions [12, 14, 31–34]. In our study we found a reduced number of adhesions after 180 days after implantation between the graft and surrounding tissues in group augmented with cells (Table 1).

Maintaining the integrity of the abdominal wall after implantation is the main factor that leads to success in wall defect reconstruction. Therefore, the material used for reconstruction must be durable enough to withstand the physiologic forces placed upon it without losing its flexibility. What is more, it should incorporate over time into the surrounding tissue. Previous studies with use of biologically derived materials have shown inadequate incorporation and tensile strength of these materials and, as a consequence, resulted in high hernia recurrence rate [35, 36]. In our study, we proved the feasibility of biologically derived type of grafts combined with cultured cells. This strength exceeds the physiologic intra-abdominal pressure values that are generated in patients [37].

Another crucial problem is the intensity of inflammation as response to implanted material. This may result in graft's acceptance or rejection. Histological examination of the cell-seeded aortic graft demonstrated only a mild inflammatory response which was comparable with those of Bellows et al. and Menom et al. [14, 38]. Decellularized wall of porcine aorta stood as a basement not only for MSC, but also for native polymorphonucleocytes and fibroblasts that deposited organized connective tissue in a manner that is consistent with natural wound healing, which is essential for effective regeneration of abdominal wall defects.

The important aspect and indication for use of so prepared porcine-vascular graft are strictly correlated with a huge number of frequent clinical implications. Our animal model is based on intraperitoneal graft placement. This situation enables us to completely assess mechanical and physical adhesion properties of the used graft. In many studies like in Jenkins et al. [39] or Lai et al. [40], it has been shown that such targeted placement is excellent to use, in a first step, especially in animal study to evaluate usefulness of selected grafting material, including cell-seeded matrices [39–41]. Probably it could have additional impact on standard extraperitoneal placement, which explained that significantly higher mechanical and physical strengths interact with intraperitoneal anatomical region compared to typical grafted extraperitoneal region. Innovative biological grafts are also an opportunity and a good alternative to standard clinical procedures for patients with mesh infections and recurrent hernia. Both situations are in some cases a significant problem which may occur in serious complications, including discharging fistulas, intra-abdominal abscess or squamous-cell carcinoma in serious infection cases, or incarceration, strangulation, and significant problem with reoperation procedures in recurrent hernia cases. Finding the ideal graft seems to be demanding but the use of such biological grafts fitted with autologous stem cells provides an opportunity to reduce the set clinical indications [42–47].

The importance of implanted biomaterial depends on the effect of strictly combined complex processes which resulted in rapid vascularization. The balance between inflammatory healing with fibroid reaction and vascularization plays the most important role in successful robust and durable biomaterial-soft tissue fusion. Our results demonstrated that MSC-seeded decellularized aortic graft showed signs of sufficient revascularization through neoangionesis after implantation. This step is crucial for proper wound healing and graft function protection from fibrosis. Moreover, vascularized graft is far better perfused with oxygen and nutrients to the repair site, enabling natural healing process or even regeneration, leading to strong incorporation of the graft into the muscle layer structure. Use of mesenchymal stem cells and aortic graft as a vehicle enabled more advanced revascularization process compared to what has been shown with other biologic matrices [14, 47–49].

In similar study authors showed good properties of acellular swine blood vessel matrix having good mechanical properties allowing fascial and vascular in-growth [14]. We have made another step by adding cells to this type of scaffold. Such combination resulted in better angiogenesis and lower inflammatory response when compared to unseeded counterpart and standard polypropylene meshes. Cell-seeded grafts have better mechanical properties than acellular grafts but worse than polypropylene mesh. Cultured mesenchymal stem cells improved mechanical and anti-inflammatory and vessel development properties of decellularized natural scaffold.

## Figures and Tables

**Table 1:** Results of tensile strength, capillary density, vessel development, inflammatory effect, and adhesions quality by Jenkins scale *n* in all three tested groups. Values are presented with standard deviations or as values range. Only results between Groups I and III in mean tensile strength are not statistically significant (*P* = 0.1).

	Group I (*n* = 7)	Group II (*n* = 7)	Group III (*n* = 7)
Mean tensile strength [N/cm]	12.7 ± 1.5	7.3 ± 1.6	14.4 ± 3.7
The average capillaries density (range)	2.7 (2.5–3)	0.7 (0.5–1)	1.5 (1.2–2)
Number of fully developed vessels (CD 31 expressions)	23 ± 3	13 ± 3	6 ± 2
Inflammatory effect (range)	0.29 (0–0.5)	1.39 (0.75–2)	2.72 (2.5–3)
Quality of peritoneal adhesions by Jenkins scale	0.29	0.71	2.71
